# Enantioselective Synthesis, Enantiomeric Separations and Chiral Recognition

**DOI:** 10.3390/molecules25071713

**Published:** 2020-04-08

**Authors:** Maria Elizabeth Tiritan, Madalena Pinto, Carla Fernandes

**Affiliations:** 1 CESPU, Instituto de Investigação e Formação Avançada em Ciências e Tecnologias da Saúde (IINFACTS), Rua Central de Gandra, 1317, 4585-116 Gandra PRD, Portugal; 2 Laboratório de Química Orgânica e Farmacêutica, Departamento de Ciências Químicas, Faculdade de Farmácia da Universidade do Porto, Rua de Jorge Viterbo Ferreira, 228, 4050-313 Porto, Portugal; madalena@ff.up.pt (M.P.); cfernandes@ff.up.pt (C.F.); 3 Centro Interdisciplinar de Investigação Marinha e Ambiental (CIIMAR), Edifício do Terminal de Cruzeiros do Porto de Leixões, Av. General Norton de Matos s/n, 4050-208 Matosinhos, Portugal

Chirality is a geometric property associated with the asymmetry of tridimensional features that accompanies our daily life at macroscopic as well as microscopic molecular levels. Chirality is a hallmark of many natural small molecules, and it is intrinsically associated with chiral building blocks as D-sugars and L-amino acids, intervening in chemical procedures of living cells, for example, as enzymes and receptors constituent proteins. Interestingly, free D-amino acids, which are naturally occurring, are important biomarkers with diagnostic value that demonstrate the importance of chiral analyses [1]. Nevertheless, the importance of chirality is recognized across many related areas as witnessed in wide-ranging fields such as chemistry, physics, biochemistry, material science, pharmacology, and many others (Figure 1). 

Though chirality has a major position in chemistry, compared with other fields, due to the importance of chiral compounds in their pure enantiomeric form, there is a need for the development of analytic methods capable of controlling the enantiomeric ratio, and to understand the behavior of chiral compounds in biological systems and in other matrices in which chirality is also present. Currently, there is a very high demand for efficient methodologies to obtain chiral bioactive compounds with a high degree of enantiomeric purity, which boosts the continuous advances in enantioselective synthesis, chiral analyses, preparative enantioseparation, as well as in chiral recognition studies. The number of publications with chirality as a subject has increased in the last decade and disclosed considerable growth in the last year, demonstrating the importance of the research in this field (Figure 2). 

These demands are related to drug discovery and development, safety in medication, food and environmental quality, materials for fine chemical industry such as chiral building blocks, among others. To meet these needs, it is essential that the international scientific community must work intensively to ensure effective production and quality of analyses of chiral compounds for a diversity of applications. For this reason, Molecules recognized the need to propose the Special Issue “Enantioselective Synthesis, Enantiomeric Separations, and Chiral Recognition”. This Special Issue is aimed at offering an opportunity to all the contributors to make their results and techniques more visible, and to present the most recent findings. This Special Issue has received remarkably positive feedback, with many contributions submitted by numerous geographically diverse scientists, resulting in a collection of 19 publications, including six exhaustive review articles [2,3,4,5,6,7], and thirteen original articles [8,9,10,11,12,13,14,15,16,17,18,19,20]. Among the contributing authors, we can find countries of origin such as Algeria, Australia, Brazil, Canada, China, Croatia, Czech Republic, Egypt, India, Italy, Japan, Portugal, Romania, Russia, and Taiwan. 

The published articles include findings related to the analytical chiral stationary phases (CSPs) for liquid chromatography (LC), currently the better choice for chiral quality control and determination of enantiomeric ratios. Faster, more efficient, and sensitive methods are urgently needed for chiral analysis, and can be achieved within small particle sizes (sub-2 µm) of the chromatographic support. The ability of the recently commercialized sub-2 µm CSP with different substituents for the fast enantioseparation of a set of drugs was demonstrated in an original article [8]. New selectors for CSPs are always required to show the response in the continuous progress of chiral analyses, and there is a need for better and low cost CSPs. In this context a new brush-Type CSP for LC was reported for enantioseparation of several drugs including nonsteroidal anti-inflammatory drugs and 3-hydroxybenzodiazepine [10]; and a new colistin sulfate CSP for nano-LC reported enantioseparation for α- and β-blockers, anti-inflammatory, antifungal, norepinephrine-dopamine reuptake inhibitors, catecholamines, sedative-hypnotic, antihistaminic, anticancer, and antiarrhythmic drugs [9]. Additionally, an exhaustive review concerning recent developments in CSPs for LC includes many different types of selectors, showing that it continues to be a field of research with great importance [2]. 

Methodologies regarding innovation in the preparative scale were also comprised in this Special Issue. For example, one article presents the purification of *R*-phenylglycinol from the phenylglycinol enantiomers by stripping crystallization, a new separation technology, which combines melt crystallization and vaporization to produce a crystalline product due to the three-phase transformation [11]. The classical preparative scale approach through diastereomeric salts formation, widely used in the pharmaceutical industry, is also presented with the resolution of 4-chloromandelic acid using the (*R*)-(+)-benzyl-1-phenylethylamine; with diastereomeric salts exhibiting significant differences in solubility and in thermodynamic properties. These differences originate from the distinct supramolecular interactions in the crystal lattice of the pair of diastereomeric salts. In addition to well-recognized hydrogen-bonding, CH/π interactions and aromatic group packing, halogen involved interactions, such as Cl…Cl and Cl/π were observed as significant contributions to the chiral discrimination [13].

The approach to achieve bioactive enantiomers by enantioselective synthesis is reported in two original publications and two reviews. One article reports the syntheses of a small library of proteomimetic quinazolinone-derived compounds and investigates their action on neurodegenerative disorders as well as the search of their potential as tumor cell growth inhibitors, giving evidence for the influence of stereochemistry on the bioactivity of diverse derivatives. Here, the enantiomeric ratio was determined by a chiral LC [17]. In another original article, the hemi-synthesis of chiral imine, benzimidazole, and benzodiazepine structures is reported by the condensation of (*S*)-(−)-perillaldehyde, the major phytochemical of the *Ammodaucus leucotrichus subsp. leucotrichus* essential oil, with different amine derivatives of 2,3-diaminomaleonitrile, *o*-phenylenediamine, and 3-[(2-aminoaryl)amino]dimedone. The chiral analyses confirm the formation of unique enantiomers and diastereomeric mixtures [16]. Small ring heterocycles, such as epoxides and aziridines, present in several natural products, are frequently involved as highly versatile building blocks in the synthesis of numerous bioactive products and pharmaceuticals. Multicomponent reactions (MCRs) have been explored in the synthesis and ring opening of these heterocyclic units. An exhaustive review about the recent advances in MCRs discuss the synthesis and applications of epoxides and aziridines to prepare other heterocycles, emphasizing the stereoselectivity of the reactions [7]. Synthesis of chiral derivatives of xanthones, an important class of bioactive compounds, as well the enantioselectivity in their biological activities, was also exhaustively revised [3]. 

Industrial production by biocatalyse using the *cis*-epoxysuccinic acid hydrolases (CESHs) was summarized, as well the perspective on the future research and applications of CESH in enantiomeric tartaric acid production [6].

Additional work concerning chiral recognition are also included in this Special Issue, such as stereochemistry assignment and chiral recognition mechanisms of sulfoxide-containing drugs [14], the structural determination of the crystal structures of three complexes between the Tiiii cavitand as host and positively charged amino acids (Arg, Lys, and His) as guests [15]; a revision concerning enantioselective drug recognition by transporters [4], and another article about enantiomeric recognition and separation by chiral nanoparticles [5]. Molecular imprinting technology is a well-established tool for the synthesis of highly selective biomimetic molecular recognition platforms. One article reports the improvement in chiral selectivity of the important β-blocker atenolol by the addition of a metal pivot *versus* the traditional molecular imprinted polymer formulation [12].

Finally, original works related to special materials as chiral liquid crystals and components for chiral sensing are presented. For the proper function of liquid crystals-based devices, not only chemical but also optical purity of materials is strongly desirable, since any impurity could be detrimental to the self-assembly of the molecules. One article demonstrated that LC with UV detection and supercritical fluid chromatography with UV and mass spectrometry detection enables full control over the chemical and enantioselectivity of the synthesis of a novel type of lactic acid-based chiral liquid crystals and the corresponding chiral building blocks [18]. Regarding chiral sensing, one article reports a path to enhanced near-field optical chirality, by means of symmetric Si nanowires arrays, which support leaky waveguide modes that enhance the near-field optical chirality of circularly polarized excitation in the shorter wavelength part of the visible spectrum, which is of interest for many chiral molecules [19]. Another article reports an enantioselective potentiometric sensor composed of a polyvinyl chloride membrane electrode modified with CC3-R porous organic cages material, used for the recognition of enantiomers of 2-amino-1-butanol [20].

This Special Issue is accessible thought the following link: https://www.mdpi.com/journal/molecules/special_issues/Chiral_separation_recognition.

As Guest Editors for this Special Issue, we would like to thank all the authors and co-authors for their contributions and all the reviewers for their effort in the careful and rapid evaluation of the manuscripts. Last but not least, we would like to appreciate the hard work done by the editorial office of the Molecules journal, as well as their kind assistance in preparing this Special Issue.

## Figures and Tables

**Figure 1:**
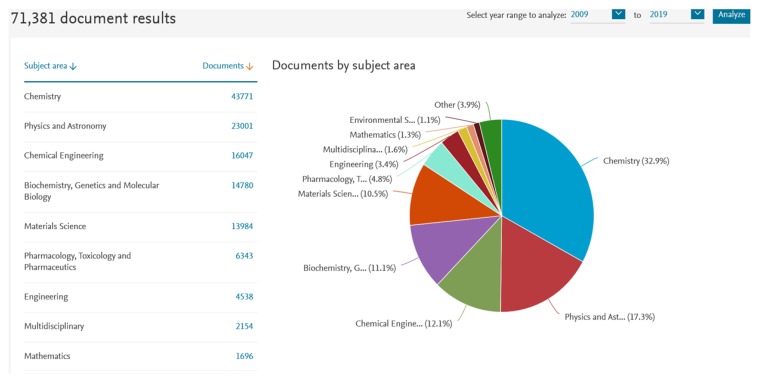
Results analysis for Scopus query “chiral” in titles, keywords, or the abstract section of articles between 2009 and 2019.

**Figure 2:**
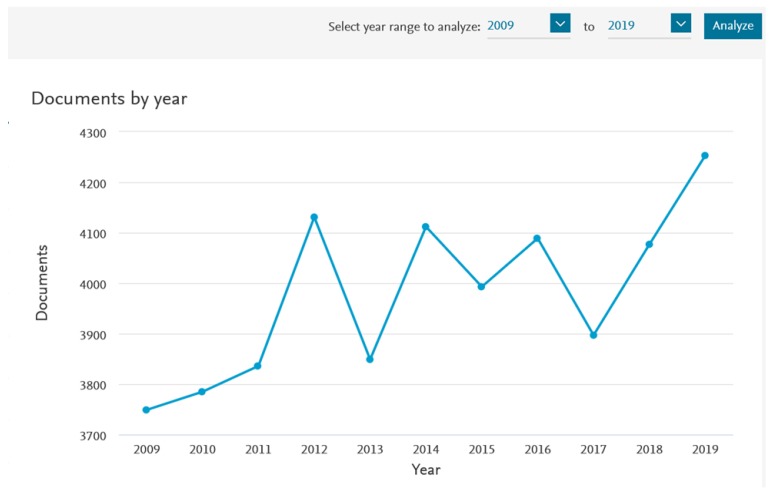
Results analysis for Scopus query “chiral” in titles, keywords, or abstract sections of articles between 2009 and 2019 (Limited to Chemistry).
